# The investigation of the role of oral-originated *Prevotella-*induced inflammation in childhood asthma

**DOI:** 10.3389/fmicb.2024.1400079

**Published:** 2024-05-28

**Authors:** Tongtong Yan, Yuling Bao, Shuyuan Cao, Ping Jiang, Zhan Zhang, Lei Li, Yulin Kang, Qian Wu

**Affiliations:** ^1^The Key Laboratory of Modern Toxicology of Ministry of Education and Department of Health Inspection and Quarantine, School of Public Health, Nanjing Medical University, Nanjing, China; ^2^Department of Respiratory Medicine, Children’s Hospital of Nanjing Medical University, Nanjing, China; ^3^Institute of Environmental Information, Chinese Research Academy of Environmental Sciences, Beijing, China

**Keywords:** asthma, oral microbiota, gut microbiota, migration, immune, lipid metabolome

## Abstract

**Background and objectives:**

The oral and gut microbiota play significant roles in childhood asthma pathogenesis. However, the communication dynamics and pathogenic mechanisms by which oral microbiota influence gut microbiota and disease development remain incompletely understood. This study investigated potential mechanisms by which oral-originated gut microbiota, specifically *Prevotella* genus, may contribute to childhood asthma etiology.

**Methods:**

Oral swab and fecal samples from 30 asthmatic children and 30 healthy controls were collected. Microbiome composition was characterized using *16S rRNA* gene sequencing and metagenomics. Genetic distances identified potential oral-originated bacteria in asthmatic children. Functional validation assessed pro-inflammatory properties of *in silico* predicted microbial mimicry peptides from enriched asthma-associated species. Fecal metabolome profiling combined with metagenomic correlations explored links between gut microbiota and metabolism. HBE cells treated with *Prevotella bivia* culture supernatant were analyzed for lipid pathway impacts using UPLC-MS/MS.

**Results:**

Children with asthma exhibited distinct oral and gut microbiota structures. *Prevotella bivia, P. disiens, P. oris* and *Bacteroides fragilis* were enriched orally and intestinally in asthmatics, while *Streptococcus thermophilus* decreased. *P. bivia, P. disiens* and *P. oris* in asthmatic gut likely originated orally. Microbial peptides induced inflammatory cytokines from immune cells. Aberrant lipid pathways characterized asthmatic children. *P. bivia* increased pro-inflammatory and decreased anti-inflammatory lipid metabolites in HBE cells.

**Conclusion:**

This study provides evidence of *Prevotella* transfer from oral to gut microbiota in childhood asthma. *Prevotella*’s microbial mimicry peptides and effects on lipid metabolism contribute to disease pathogenesis by eliciting immune responses. Findings offer mechanistic insights into oral-gut connections in childhood asthma etiology.

## Highlights

- *Prevotella* migrating from oral cavity to gut become the driver of childhood asthma.
- Microbial mimicry peptides secreted by *Prevotella* would trigger self-immune effect to induce asthma.
- *Prevotella* would exacerbate inflammation in childhood asthma by induing immune-related lipid metabolites in host.

## 1 Introduction

Oral microbiota plays a crucial role in the human microbial community, consisting of more than 700 species (Palmer, 2000). Due to its easy accessibility for sampling and its connection to overall health, the oral microbiome has become an integral part of the human microbiome program (HMP). The major phyla found in oral microbiota include Firmicutes, Bacteroidetes, Proteobacteria, Actinobacteria and Fusobacteria (Dewhirst et al., 2010). Research has demonstrated that the imbalance in the oral microbiota community is associated not only with local oral diseases but also with respiratory, cardiovascular and neoplastic conditions (Genco and Van Dyke, 2010; Le Bars et al., 2017). For example, lower level of *Lactobacillus* in oral microbiota during the first seven years after birth is closely linked to increased risk of developing of allergic conditions (Dzidic et al., 2018). Additionally, elevated levels of *Moraxella* and *Haemophilus* in sputum samples have been correlated with neutrophilic asthma (Durack et al., 2017). It is worth noting that oral microbiota is not solely colonized in the oral cavity; they can migrate to other body sites, leading to local inflammatory reaction and infection. Recent research has highlighted the correlation between the oral and gut microbiota. For example, oral administration of *Porphyromonas gingivalis* has been shown to disrupt the gut microbiota community, characterized by an increase in Bacteroidetes, a decrease in Firmicutes, and elevated serum endotoxin level, ultimately triggering gut inflammation (Kato et al., 2018). *Streptococcus salivarius*, an early colonizer in the oral cavity, is also capable of colonizing the gut mucosa. Notably, it down-regulated the expression of nuclear transcription factor NF-кB in human intestinal cells, which plays an important role in intestinal inflammatory response and homeostasis (Couvigny et al., 2015). Meanwhile, maintaining gut microbiota homeostasis is essential for lung health. Communication between the gut and lungs occurs via the gut-lung axis. For example, a decrease in *Bifidobacteria* and an increase in *Clostridium* in the gut have been associated with early-life asthma (Arrieta et al., 2015). Furthermore, animal experimental studies have demonstrated that germ-free mice are more susceptible to lung diseases and allergic inflammation (Simonyte Sjödin et al., 2016). The effect of gut microbiota on mucosal immunity is not only restricted to the gastrointestinal tract; it extends to the bronchial epithelium and lymphoid tissue as well, thereby affecting certain inflammatory diseases (Yuan et al., 2022). T and B cells induced by intestinal lamina propria can migrate through the lymphatic system, inducing immune responses (Enaud et al., 2020; Zhang et al., 2020). Probiotics, such as *Lactobacillus* and *Bifidobacteria*, which have been shown in mouse models to regulate T cell responses and suppress allergic reactions (Wang et al., 2017; Pyclik et al., 2021). The shared genera that coexist in both oral microbiota and gut microbiota have become a subject of interest in studying the potential role of oral-originated gut species in diseases. Hu et al. (2023) found that oral microbiota from alcohol dependence can colonize ectopically in the intestines. Recent investigations have indicated a translocation and migration of specific oral taxa into the gut of COVID-19 patients (Wu et al., 2021). Certain bacteria originated from the oral cavity may influence the development and health of the immune system, potentially contributing to the development or exacerbation of inflammatory diseases such as cardiovascular disease and rheumatoid arthritis (Hajishengallis, 2015; Graves et al., 2019). During this process, microbial-derived mimic antigen epitopes are activated, promoting the imprinting of specific T helper cells and the accumulation of inflammatory cells, thereby influencing systemic immune responses (Chen et al., 2021).

Additionally, metabolites derived from gut microbiota, such as short-chain fatty acids (SCFAs), are crucial signaling molecules for maintaining immune homeostasis. These SCFAs can enter the circulatory system via the lymphatic system and participate in regulating immune responses in the lungs, providing beneficial effects in alleviating asthma and chronic obstructive pulmonary diseases (Roduit et al., 2019). Bacterial-derived inflammatory mediators were associated with an increased risk of atopy and asthma. For example, an elevated concentration of the gut microbiota-derived metabolite 12,13-diHOME in infant feces was linked to an increased risk of atopy and asthma in childhood. This metabolite was produced by *Bifidobacterium* and *Faecalibacterium* which could promote allergic inflammation by reducing the frequency of lung Treg populations and *IL-10* production (Fujimura et al., 2016; Levan et al., 2019).

Based on these clues, we aimed to elucidate the communication between oral microbiota and gut microbiota and investigate the potential mechanism of the effects of oral-originated gut microbiota on childhood asthma in the present study. *16S rRNA* gene sequencing and metagenome sequencing were used to analyze the alterations in oral and gut microbiota in a case–control study. Genetics analysis was used to identify key oral-originated gut microbiota in the children with asthma. Metabolomic analysis and *in vitro* experiments was used to explore the potential mechanism.

## 2 Methods

### 2.1 Samples

In case–control study, 30 children with asthma and 30 controls without allergic diseases were recruited from Nanjing Children’s hospital affiliated to Nanjing Medical University and Jiangsu Province Hospital of Chinese medicine. Covariates were considered in this study including age, sex (male vs. female), BMI (body mass index), passive smoking (yes vs. no). Inclusion criteria: The children with asthma were diagnosed by physicians without taking antibiotics during the relief stage in recent three months. The control group did not have any respiratory system-related diseases or without allergic disease. Exclusion criteria: (1) subjects with recent surgical history were excluded; (2) subjects with recent Oral problems were excluded; (3) subjects with underlying diseases were excluded; (4) subjects with overweight or obesity were excluded. The demographic information of the children with asthma in the study was in Supplementary Table S1. Oral swabs and fecal samples were collected and stored at −80°C for further experiments. Blood samples were collected from control individuals. Peripheral blood mononuclear cells (PBMCs) were isolated using human lymphocyte separation medium (Fcmacs biotech co., ltd.). This study was approved by the ethics Committee of Nanjing Medical University [approval no. Nan med Univ ethical review (2019)02080-1]. Written informed consent was obtained from all guardians.

### 2.2 *16S rRNA* gene sequencing

Total genomic DNA was extracted from oral swabs and fecal samples according to the E.Z.N.A.® soil kit (Omega Biotek, Norcross, GA). The V3-V4 variable region of the bacterial *16S rRNA* gene was amplified by PCR, using the forward primer 338F (5’-ACTCCTAC GGGAGGCAGCAG-3′) and reverse primer 806R (5’-GGACTACHVG GGTWTCTAAT-3′). The PCR amplification system included 4 μL 5 × FastPfu buffer, 2 μL 2.5 mM dNTPs, 0.8 μL of each primer (0.5 μM), and 10 ng DNA template. PCR conditions were as following: 95°C denaturation for 3 min, 27 cycles (95°C for 30 s, 55°C for 30 s and 72°C for 45 s), finally 72°C for 10 min. The PCR products were purified and recovered by AxyPrep DNA Gel Extraction Kit (Axygen Biosciences, Union City, CA, USA). Sequencing was performed using the Miseq PE300 platform (Illumina, San Diego, USA). The raw sequences were quality controlled and assembled using Trimmomatic and FLASH software. The operational taxonomic units (OTUs) were clustered based on 97% similarity using UPARSE (version 7.1^1^) software, and the OTUs sequences were annotated using the RDP classifier^2^ software. The free online platform of the Majorbio I-Sanger Cloud Platform (Shanghai Majorbio Bio-pharm Technology Co., Ltd)^3^ was used for data analysis.

### 2.3 Metagenomics sequencing

Metagenomic sequencing was performed on the Illumina NovaSeq platform (Illumina, USA). The raw reads from metagenome sequencing were used to generate clean reads using the fastp^4^ on the free online platform of Majorbio Cloud Platform.^5^ These high-quality reads were then assembled to contigs using MEGAHIT,^6^ which makes use of succinct de Bruijn graphs. Contigs with the length being or over 300 bp were selected as the final assembling result. Open reading frames (ORFs) in contigs were identified using MetaGene.^7^ Representative sequences of non-redundant gene catalog were annotated based on the NCBI NR database using blastp as implemented in DIAMOND v0.9.19 with e-value cutoff of 1e-5 using Diamond^8^ for taxonomic annotations. Functional differences were found by LEfSe.

### 2.4 Strain-level analysis based on the genetic distance

The contigs, which have been assembled, were aligned with the reference gene sequences of NCBI strains using Blast software (version 2.9.0). Sequences with a similarity ≥80% and a length ≥ 200 bp were selected. The selected sequences were then subjected to gene annotation using Prokka software (version 1.13). The annotation results for the sequences identified as *16S rRNA* gene were further aligned using the MUSCLE method in MEGA software (version 11.0.13), and the genetic distance was calculated using the p-distance method.

### 2.5 Functional analysis of microbial mimicry peptides

Epitopes extracted from the Immune Epitope Database (IEDB)^9^ were identified as triggers for immune reaction in asthma. These epitopes’ amino acid sequences were then compared to the protein sequences of species. Only epitopes with a minimum identity and coverage of 80% were chosen for subsequent experimental investigation. These microbial mimicry peptides were synthesized by Nanjing Branch of GenScript Biotech Company. PBMCs were isolated from anticoagulated blood using human lymphocyte separation fluid and re-suspended in R1640 complete medium (Zhang et al., 2021). Peptide segments were added to the cells for stimulation at a final concentration of 40 μg/mL, and the cells were cultured at 37°C and 5% CO_2_ for 48 h before collection. Total RNA was extracted from the cells using the TRIzol method (Tiangen, China), and the concentration was measured using a nucleic acid protein analyzer (Thermo NanoDrop 2000, US). Reverse transcription was performed using the HiScript II Q RT SuperMix kit (Vazyme Biotech) following the instructions provided. Reverse-transcription quantitative polymerase chain reaction (RT-qPCR) was used to quantify asthma-related inflammatory factors *IL-4, IL-5, IL-6, IL-8, IL-13*, and *IL-17A*. The PCR system consisted of cDNA, primer (0.2 μmol/L) and ChamQ Universal SYBR qPCR Master Mix (5 μL), with a total system volume of 10 μL. The primers were synthesized by Nanjing Branch of GenScript Biotech Company, with the following sequences: For *IL-4*, the forward primer was 5’-CGGCAACTTTGTCCACGGA-3′, and the reverse primer was 5’-TCTGTTACGGTCAACTCGGTG-3′; for *IL-5*, the forward primer was 5’-GGAATAGGCACACTGGAGAGTC-3′, and the reverse primer was 5’-CTCTCCGTCTTTCTTCTCCACAC-3′; for *IL-6*, the forward primer was 5’-CCTTCGGTCCAGTTGCCTTCTC CCT-3′, and the reverse primer was 5’-GGGCTGAGATGCCGTCGA GGATGTA-3′; for *IL-8*, the forward primer was 5’-CCACCGGAAG GAACCATCTC-3′, and the reverse primer was 5’-TTCCTTGGG GTCCAGACAGA-3′; for *IL-13*, the forward primer was 5′- CCTCATGGCGCTTTTGTTGAC-3′, and the reverse primer was 5′- TCTGGTTCTGGGTGATGTTGA-3′; for *IL-17A*, the forward primer was 5’-AGATTACTACAACCGATCCACCT-3′, and the reverse primer was 5’-GGGGACAGAGTTCATGTGGTA-3′; for the reference gene *β-actin*, the forward primer was 5’-CATGTACGTTGC TATCCAGGC-3′, and the reverse primer was 5’-CTCCTTAATGTCA CGCACGAT-3′. According to the instructions of the kit, PCR reactions were performed in a LightCycler® 96 detection system (Roche Diagnostics (Shanghai) Ltd., Switzerland). The amplification parameters were as follows: an initial denaturation step at 95°C for 3 min, followed by 45 cycles of denaturation at 95°C for 10 s, annealing at 60°C for 30 s, and extension at 72°C for 10 s, with a final extension step at 60°C for 60 s. The melting curve was set according to the instrument’s standard. For data analysis, *β-actin* was used as the reference gene for normalization, and triplicate measurements were performed. The relative expression levels of the target genes were calculated using the 2^-ΔΔCt^ method.

### 2.6 Non-targeted metabolomic analysis of fecal samples

Fifty milligrams of feces were placed into a 1.5 mL Eppendorf tube, followed by the addition of 1 mL of pre-chilled ultrapure water and magnetic beads. The mixture was then ground at 30 Hz for 3 min at 4°C. The quality control sample was prepared by mixing 50 μL of each individual sample. The sample, along with the quality control mixture, was then centrifuged at 4°C and 12,000 rpm for 15 min, and the supernatant was collected. The remaining pellet was retreated according to the above steps. The above supernatant was mixed well and then centrifuged at 4°C and 12,000 rpm for 15 min. Finally, 200 μL of the supernatant was taken for further analysis.

For the metabolomic analysis, the method was well established in our laboratory. Briefly, a Dionex Ultimate 3,000 UPLC (Dionex, Germering, Germany) combined with a Q-Orbitrap-MS system equipped with a heated electrospray ionization source (Q-Exactive plus MS, Thermo Scientific, MA) was used. The column was a C_18_ column (100 mm × 2.1 mm, 1.9 μm), and the column temperature was set at 40°C. The mobile phase solvents, solvent A (0.1% formic acid-water) and solvent B (0.1% formic acid-acetonitrile), were used for sample elution. The column was washed with 100% solvent B and equilibrated with 100% solvent A. The flow rate was 0.2 mL/min, and the injection volume was 5 μL. The MS parameters were as follows: sheath gas flow rate of 10 arbitrary units, spray voltage of 3.0 kV, capillary temperature of 320°C, and auxiliary gas heater temperature of 30°C. A heated electrospray ionization source was used, and the samples were detected in full scan mode. Data for samples in the positive ion mode and negative ion mode were collected in the range of 30–800 m/z.

The raw data was processed using the MSConvert software to convert it to the RAW format. The R software was used for normalization, data transformation, centering, and standardization. Then, the data was imported into the online database XCMS Online Metabolomics (Scripps Research Institute, USA). After exporting the data, a pre-selection was performed based on the criteria of metabolite features >50% and relative standard deviation (RSD) ≤30%. Subsequently, the R software (version 3.5.1) was used for principal component analysis (PCA) to simplify and reduce the dimensionality of the data. The overall distribution between sample groups was analyzed using distance algorithms. The variable importance in the projection (VIP) was used as an important parameter to indicate disruptions in the body’s metabolic network. Candidate variables were selected based on VIP ≥1.0 and statistical significance (adjusted *p*-value <0.05). Metabolites were compared with the HMDB Library^10^ based on positive and negative ion modes (M + H and M-H).

### 2.7 HBE cells treated with the supernatant of *Prevotella bivia*

Human bronchial epithelial (HBE) cells were generously provided by Dr. Haiyan, Chu from the School of Public Health, Nanjing Medical University. HBE cells were cultured in DMEM (Gibco, USA) containing 10% FBS (TransGen Biotech Co., Ltd., Beijing, China) supplemented with 1% penicillin–streptomycin at 37°C with 5% CO_2_. *Prevotella bivia* (ATCC 29303) and modified reinforced clostridial broth (ATCC Medium 2,107) were purchased from Mingzhou Biotechnology Co., LTD. The strain was cultured in Medium 2,107 at 37°C in the Bugbox anaerobic system (Bug Box, Ruskinn Technology, Leeds, UK) for 48 h. The cultures were centrifuged at 5,000 rpm for 5 min at 4°C. The supernatant was filtered to remove bacteria. HBE cells were treated for 24 h either 10% of bacterial supernatant or ATCC Medium 2,107.

### 2.8 Analysis of lipid metabolic profiles by UPLC-MS/MS

HBE cells were washed twice with PBS and resuspended in 100 μL ultrapure water extraction solution (containing protease inhibitors, PMSF, and EDTA). Five hundred microliter of lipid extraction solution and 50 μL cell suspension were mixed by vortexing for 2 min. The mixed samples were sonicated for 5 min, added with 100 μL water, vortexed for 1 min, and then centrifuged at 4°C and 12,000 rpm for 10 min. The 200 μL supernatant was collected and dried with nitrogen. The dried supernatant was re-dissolved in 200 μL of isopropanol/acetonitrile (50%/50%) for further analysis. Metabolic data were acquired by two data acquisition systems, i.e., ultra-performance liquid chromatography (UPLC, ExionLC™ AD) and tandem mass spectrometry (MS/MS, QTRAP® 6,500+). The samples were separated on a Thermo Accucore™ C30 column (2.6 μM, 2.1 mm × 100 mm i.d.) at a flow rate of 0.35 mL/min, and the temperature of 45°C. The solvent system was as follows: A: 0.1% formic acid and 10 mmol/L ammonium formate in acetonitrile/water (60%/40%); B: 0.1% formic acid and 10 mmol/L ammonium formate in acetonitrile/isopropanol (10%/90%). The samples were detected in full scan mode. The characteristic ions of each lipid metabolite were processed by multiple reaction monitoring. Lipid metabolites were identified by MWLDB (Metware lipidomics database v 3.0) provided by MetWare, Co., Ltd.^11^ (Wuhan, China). The semiquantitative analysis of the metabolites were calculated based on the peak area ratio of the analyte to the internal standard (Supplementary Table S2).

### 2.9 Statistical analysis

Wilcoxon’s rank sum tests and student’s *t* tests were used to determine the significance of differences in alpha diversity, composition, genetic distance and inflammatory factor expression between two groups. The aforementioned tests were carried out by utilizing either R version 3.5.1 or GraphPad Prism version 8.0. A two-sided *p*-value of less than 0.05 was considered statistically significant.

## 3 Results

### 3.1 The alteration of the microbial diversity of oral and gut microbiota in the children with asthma

In this study, the mean (± SD) of age of children with asthma and the control was 6.52 ± 2.60 years and 6.06 ± 2.78 years, respectively; the number of males and females in the group with asthma was 14:16 and 18:12 in the control group. There was no significant difference in the distribution of age and sex between two groups. BMI of all subjects was all in the normal range. All of subjects reported without passive smoking.

**Figure 1:**
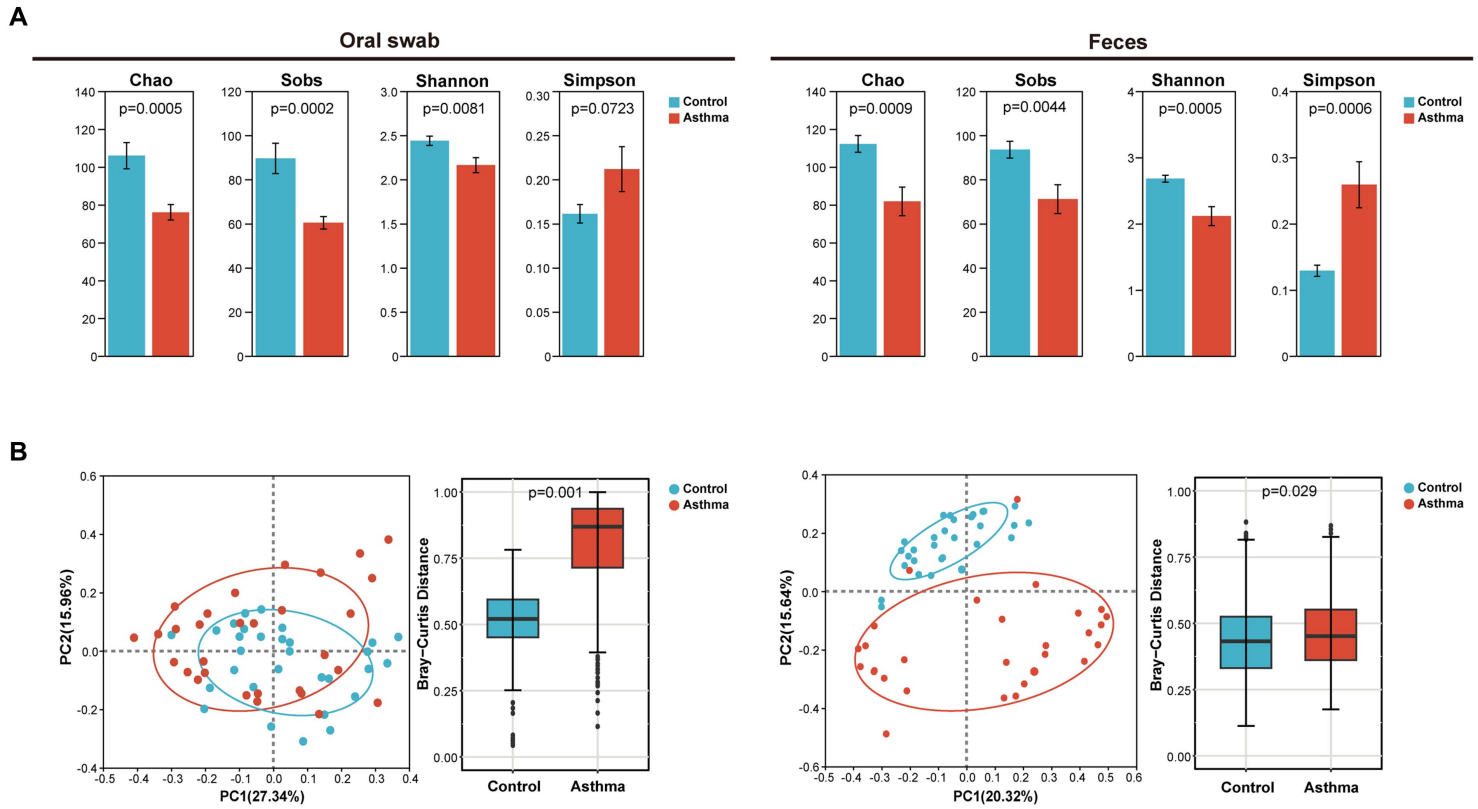
The changes in microbial diversity in oral and gut samples of the children with asthma. **(A)** Chao, sobs, shannon and simpson index of oral microbiota and gut microbiota in subjects with or without asthma. **(B)** PCoA and Bray-Curtis distance of oral and gut microbiota. All microbiota was analyzed using *16S rRNA* gene sequencing. *n* = 30 for oral swabs and 30 for feces. Student’s *t*-test was used for statistical analysis in **(A)** and permutational multivariate analysis of variance in **(B)**.

The oral microbiota and gut microbiota of both the control and the children with asthma were analyzed through *16S rRNA* gene sequencing. Compared to the control group, the Chao, Sobs and Shannon indices of oral microbiota and gut microbiota in the children with asthma were significantly decreased, and the Simpson index increased (Figure 1A). Beta (β) diversity analysis using PCoA (Principal co-ordinates analysis) was used to visualize the differences among groups. Bray-Curtis distance demonstrated statistically significant differences in β diversity of oral microbiota and gut microbiota between two groups (Figure 1B).

### 3.2 The changes in the composition of oral and gut microbiota in the children with asthma

**Figure 2:**
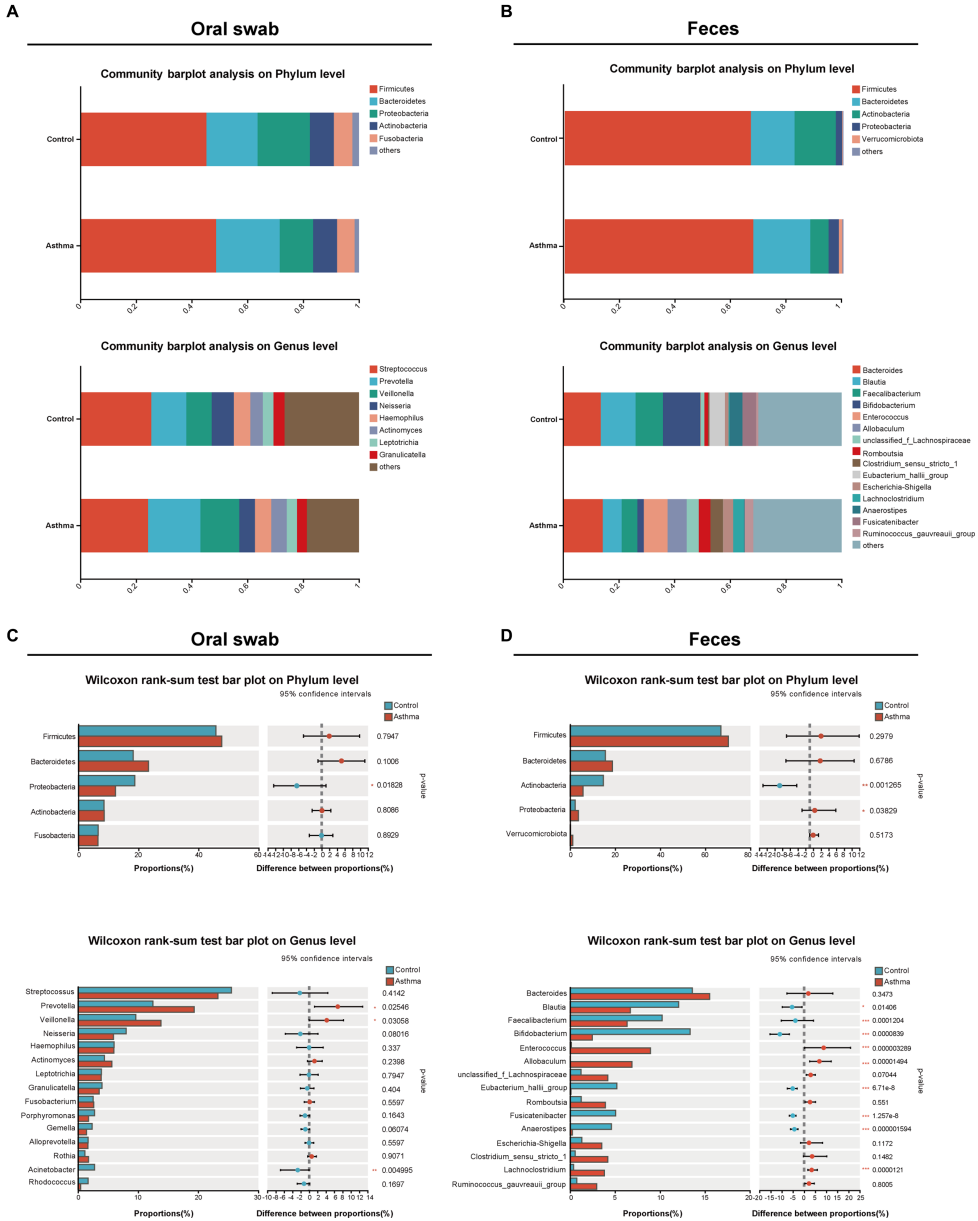
The alterations of oral and gut microbial composition in the children with asthma. Bar plots showing relative abundances of oral microbiota **(A)** and gut (fecal) microbiota **(B)** microbiota at phylum level and genus level in subjects with or without asthma. The distribution of microbial communities in each group was displayed using mean values. The comparison of the phyla and genera in oral microbiota **(C)** and gut (fecal) microbiota **(D)** between two groups. Wilcoxon rank-sum test was used for statistical analysis. *p < 0.05, **p < 0.01, ***p < 0.001.

The dominant bacterial phyla in oral microbiota were Firmicutes, Bacteroidetes, Proteobacteria, Actinobacteria, and Fusobacteria, while the top eight dominant bacterial genera were *Streptococcus*, *Prevotella*, *Veillonella*, *Neisseria*, *Haemophilus*, *Actinomyces*, *Leptotrichia* and *Granulicatella* (Figure 2A); the dominant phyla of gut microbiota were Firmicutes, Bacteroidetes, Actinobacteria, Proteobacteria, and Verrucomicrobiota; the top eight dominant genera were *Bacteroides*, *Blautia*, *Faecalibacterium*, *Bifidobacterium*, *Enterococcus*, *Allobaculum*, *Romboutsia*, and *Clostridium_sensu_stricto_1* (Figure 2B), which were consistent with previous studies (Hu et al., 2022; Zheng et al., 2022).

In oral microbiota, the relative abundance of Proteobacteria was significantly decreased, and although the relative abundance of Firmicutes and Bacteroidetes was increased, there was no statistical significance in the children with asthma. At the genus level (relative abundance >5%), the relative abundance of *Prevotella* and *Veillonella* was significantly increased, and *Streptococcus* showed a decreased trend in the children with asthma (Figure 2C). In gut microbiota, there was an increase in the relative abundance of Firmicutes and Bacteroidetes without significance, and the relative abundance of Proteobacteria was significantly increased and Actinobacterioat was significantly decreased in the children with asthma. At the genus level (relative abundance >5%), the relative abundance of *Enterococcus* and *Allobaculum* was significantly increased, while the relative abundance of *Blautia*, *Bifidobacterium*, *Faecalibacterium*, *Eubacterium_hallii_group*, and *Fusicatenibacter* was significantly decreased in the children with asthma (Figure 2D).

### 3.3 The enrichment of oral-originated microbiota in the gut of children with asthma

**Figure 3:**
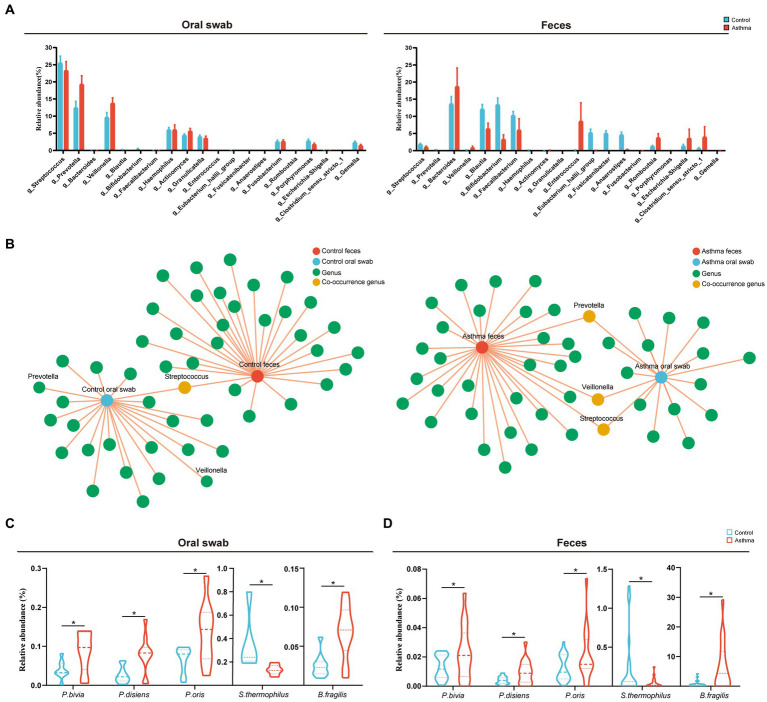
Shared microbiota in the oral cavity and gut of the children with asthma. **(A)** Relative abundances of the top 20 shared genera in subjects with or without asthma. **(B)** Co-occurrence network analysis of oral and gut microbial genera in patients with asthma and healthy controls. **(C,D)** The comparison of the relative abundance of *Prevotella bivia*, *Prevotella disiens*, *Prevotella oris, Streptococcus thermophilus* and *Bacteroides fragilis* in oral and gut microbiota between two groups. *n* = 10 in the control and the children with asthma, respectively, for microbiota metagenomic sequencing. *p < 0.05, Wilcoxon rank-sum test.

One hundred and twenty-one shared OTUs coexisted in oral and gut microbiota shown in the Venn diagram (Supplementary Figure S1). The relative abundance of the top 20 shared genera showed the relationship between oral and gut microbiota (high in oral and low in gut microbiota, vice versa) (Figure 3A). Furthermore, we conducted a co-occurrence network analysis on the distribution patterns between samples and species. In the control group, only *Streptococcus* was present in both the oral cavity and gut, while *Prevotella* and *Veillonella* were exclusively found in the oral cavity. In the group with asthma, *Prevotella*, *Veillonella*, and *Streptococcus* were observed in both oral cavity and gut (Figure 3B).

**Figure 4:**
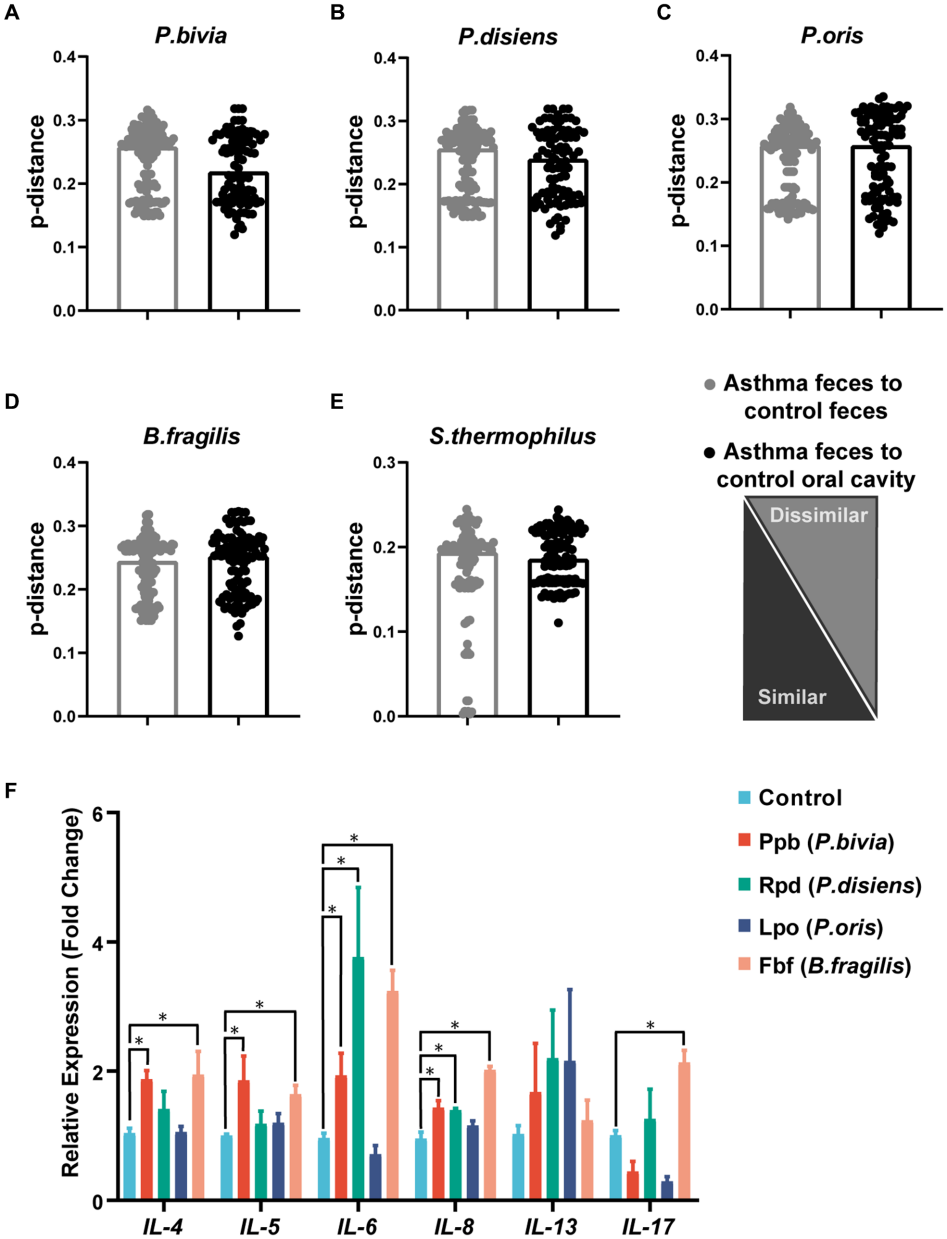
Genetic distance analysis of asthma-enriched species and functional validation of microbial peptide molecular mimicry. Indices of intrasubject similarity (p-distance) between the bacterial communities of the two source sites (oral cavity and gut) and the target site (gut) of that subject. As shown, smaller distances mean greater similarities between the microbial communities of the samples indicated **(A–E)**. After stimulation with different peptides, the mRNA transcription levels of *IL-4, IL-5, IL-6, IL-8, IL-13*, and *IL-17A* in PBMCs were measured (*n* = 4). *p < 0.05, student’s *t* test was used for statistical analysis **(F)**.

Further analysis of microbiota metagenomic sequencing results revealed a significant increase in the abundance of *P. bivia*, *P. disiens*, and *P. oris* in both oral cavity and gut. Additionally, there was a significant increase in the relative abundance of *Bacteroides fragilis* in the oral and gut microbiota of children with asthma, while the relative abundance of *Streptococcus thermophilus* was significantly decreased (Figures 3C,D). To confirm the oral-gut migration of these species in the children asthma, we conducted a strain-level analysis based on *16S rRNA* gene using oral and fecal metagenome of the control and fecal metagenome of the group with asthma. Genetic distance of *16S rRNA* gene was calculated by p-distance. The strains of *Prevotella bivia*, *P. disiens*, *P. oris* and *S. thermophilus* in the gut of the group with asthma had a closer relationship to those in the oral cavity of the control, indicating these species originated from the oral microbiota (Figures 4A–E).

### 3.4 Microbial mimicry peptides from the enriched microbiota in the gut induced the expression of inflammatory factors

Through aligning the amino acid sequences of *P. bivia*, *P. disiens*, *P. oris*, and *B. fragilis* with the IEDB database, microbial mimicry peptides that exhibited more than 80% similarity to asthma antigenic epitopes were selected (Supplementary Table S3). These peptides were used to stimulate PBMCs, and the expression of relevant inflammatory factors was subsequently evaluated. The results showed that Ppb from *P. bivia* induced *IL-4, IL-5, IL-6,* and *IL-8* expression; Rpd from *P. disiens* induced *IL-6* and *IL-8* expression. Fbf was derived from *B. fragilis*, and induced *IL-4, IL-5, IL-6, IL-8*, and *IL-17A* expression (Figure 4F).

### 3.5 Gut microbiota interacted with fecal metabolome related to childhood asthma

**Figure 5:**
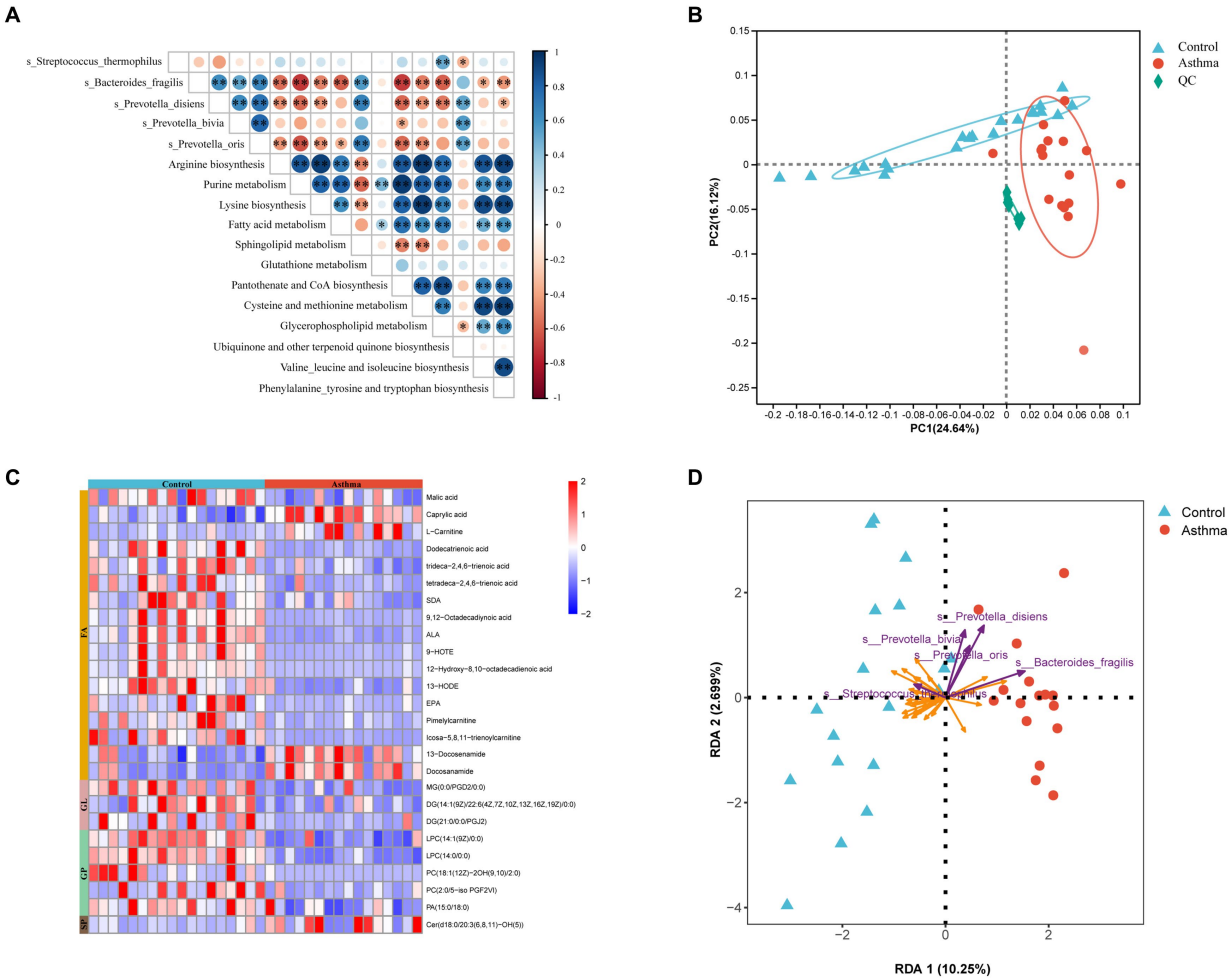
Correlations between asthma-related microbial species and metabolites in feces. **(A)** Heatmap representation of spearman correlation between microbial species and their corresponding KEGG pathways. *p < 0.05, **p < 0.01, Spearman. **(B)** PCA showed alterations of fecal metabolome in the children with asthma. **(C)** Heatmap analysis of differential lipid metabolites. Red color represents upregulated metabolites while blue color represents downregulated metabolites. **(D)** Redundancy analysis (RDA) of the variable relationships between differential microbiota and lipid metabolites. The purple arrows represent bacteria, and the orange arrows represent lipid metabolites. FA, Fatty Acyl; GL, Glycerolipids; GP, Glycerophospholipids; SP, Sphingolipids.

Based on gut metagenomic data, we identified KEGG pathways with an LDA (Linear discriminant analysis) score greater than 2 by LEfSe analysis (Supplementary Figure S2). The asthma group was characterized by amino acid metabolism and lipid metabolism. Spearman correlation analysis was performed between the above 5 different species and selected pathways. *B. fragilis, P. bivia*, *P. disiens* and *P. oris* had associations with these two types of metabolic pathways (Figure 5A).

In order to elucidate the functional changes in gut microbiota and their interaction with the host, we conducted a comprehensive analysis of the metabolome in fecal samples using an untargeted ultrahigh-performance liquid chromatography-mass spectrometry (UPLC-MS) approach. The score plots generated from PCA depicted distinct separation between the control and the group with asthma (Figure 5B). The differential metabolism were determined based on a variable importance in projection (VIP) score greater than 1 and a false discovery rate lower than 0.05. The certain metabolism such as malic acid, SDA, ALA, and EPA (fatty acyl); DG and MG (glycerolipids); LPC, PC, and PA (glycerophospholipids) were decreased, while L-carnitine (fatty acyl) and Cer (sphingolipids) were increased compared to the control (Figure 5C; Supplementary Table S4).

Next, a redundancy analysis (RDA) using metagenomic and metabolomic data revealed a complex co-occurrence pattern and correlation between bacteria associated with childhood asthma and characterized metabolites from the essential metabolic pathways. In the gut of the children with asthma, *P. bivia*, *P. disiens,* and *P. oris* showed a negative correlation with glycerophospholipid (LPC, PC, and PA), and *B. fragilis* showed a positive correlation with L-carnitine and Cer. As a probiotic, *S. thermophilus* showed an opposite trend compared to *Prevotella* and *Bacteroides* (Figure 5D).

### 3.6 *Prevotella bivia* affected lipid metabolism in HBE cells

**Figure 6:**
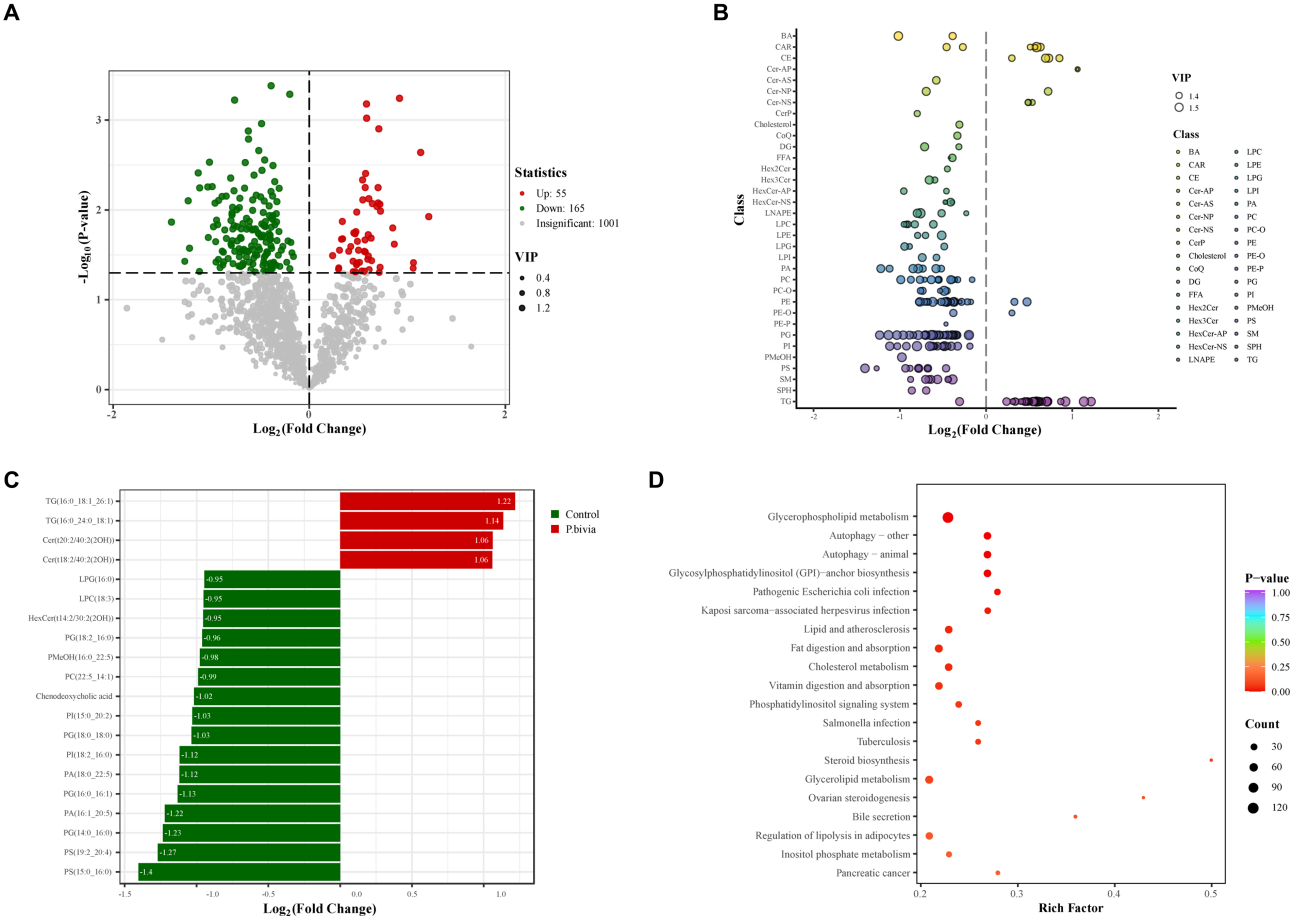
The disturbance of lipid metabolism in HBE cells treated with the supernatant of *Prevotella bivia*. **(A)** The volcano plot analysis. The criteria were VIP > 1 and *p*-value <0.5. The red (upregulated) and green (downregulated) dots represent lipid metabolites with significant differences, while gray dots indicate lipid metabolites with no significant differences. **(B)** The scatter plot illustrated the distribution of differential lipids in subclasses. The color of each circle represented different lipid subclasses, and the size of them indicated the VIP. **(C)** The bar chart showed the top twenty differential lipids in terms of fold change. **(D)** Enrichment of different lipid metabolites (*n* = 3).

Human bronchial epithelial (HBE) cells were cultured with the supernatant of *P. bivia* and were detected with lipid metabolome based on UPLC-MS/MS. PCA performed on all the samples revealed that a clear clustering of QC (quality control) samples, and lipid components in the control group and treatment group were well separated (Supplementary Figure S3). A total of 1,221 lipid metabolites were detected in the samples, categorized into 45 lipid subclasses, and the types and quantities of lipid subclasses detected were presented in Supplementary Figure S4A. The distribution of various lipid subclasses between the two groups was shown in Supplementary Figure S4B. Based on volcano plot analysis, 220 differential lipid metabolites were selected with VIP > 1 and adjusted *p*-value <0.05. Among them, 55 showed a significant increase, while 165 showed a significant decrease in the treatment group (Figure 6A). Through visualizing the distribution of differential lipids in different lipid subclasses (Supplementary Figure S5) and filtering the top 20 based on fold change, in the treatment group, it revealed that a significant decrease in LPC, LPG, PA, PC, PG, PI, and PS, and a significant increase in CE, Cer, and TG (Figures 6B,C). These results were partially consistent with findings from previous non-targeted metabolomics analysis. Most of lipid metabolites (87.72%) were enriched in metabolic pathways (Supplementary Figure S6). Based on the differential lipid metabolites, KEGG pathway enrichment analysis revealed pathway disruptions in the treated group, primarily enriched in glycerophospholipid metabolism (*p* < 0.05) (Figure 6D).

## 4 Discussion

Several studies have suggested a potential link between alterations in oral and gut microbial composition and an increased risk of childhood asthma (Fujimura et al., 2016; Durack et al., 2020). However, there is limited research focusing on direct evidence of migration between oral microbiota and gut microbiota in the children with asthma. In the present study, we found that *Prevotella* can migrate from the oral cavity to the gut and influence childhood asthma by secreting microbial mimicry peptides and regulating lipid metabolites associated with inflammation.

Consistent with previous results, *16S rRNA* sequencing data showed that a significant decrease in alpha diversity of both oral microbiota and gut microbiota in the children with asthma (Patrick et al., 2020; Hu et al., 2022). In the oral microbiota of the children with asthma, there was a significant increase in the abundance of *Prevotella* and *Veillonella*. They are common commensals in the oral cavity, the increase of which has been reported to lead to the recruitment of monocytes and T cells chemokines, consequently increasing the risk of asthma (Thorsen et al., 2019). Metagenome sequencing revealed that *P. bivia, P. disiens, P. oris* and *B. fragilis* were increased and *Streptococcus thermophilu* was decreased in the gut of the children with asthma. *P. bivia, P. disiens,* and *P. oris* may be considered to originate from the oral cavity and transmit to the gut by genetic distance analysis. Some studies have demonstrated that *Prevotella* as key members of the oral microbiota were found in the gut of patients and associated with certain gastrointestinal disorders (Schmidt et al., 2019). *Prevotella* can mediate mucosal inflammation. *Prevotella* can active mucosal Th17 immune responses and neutrophil recruitment, further leading to systemic dissemination of inflammatory factors and bacterial products, and in turn may affect systemic disease outcomes, including asthma (Larsen, 2017). *B. fragilis* is a major colonizer of the human gut where *Bacteroides* is a predominant genus. *B. fragilis* colonized in feces at the age of 3 weeks increased the risk of developing asthma later on Vael et al. (2008). In mouse models, the colonization of *B. fragilis* in the gut led to impaired intestinal barrier function, increased secretion of IL-17 in the lungs during allergic airway inflammation, and oxidative stress (Wilson et al., 2023). Additionally, *S. thermophilus* in the gut of the children with asthma was more closely related to the oral cavity in genetics. What’s more, the relative abundance of *S. thermophilus* was reduced in both locations in the group with asthma. *S. thermophilus* is a probiotic that has anti-allergic effects and can reduce the production of allergen-specific IgE (Peng et al., 2007). The extracellular polysaccharides produced by *S. thermophilus* can protect the integrity of the intestinal barrier and reduce the expression of pro-inflammatory cytokines, playing an important role not only in gastrointestinal disease but also in other inflammatory disease prevention (Mizuno et al., 2020). For example, in the rat experiment of osteoarthritis, *S. thermophilus* can reduce synovial tissue inflammation (Lin et al., 2021).

Recently several studies have indicated that microbial mimicry peptides may trigger an immune response by activating autoimmune T cells and B cells as mimic autoantigens in some diseases (Chen et al., 2017). Five of microbial mimicry peptides derived from three *Prevotella* species and *B. fragilis* were capable of stimulating PBMCs to express *IL-4, IL-5, IL-6, IL-8,* and *IL-17A* genes. Rpd from *P. disiens* and Fbf from *B. fragilis* were similar to the *Blattella germanica* antigen which can be presented to T cells stimulating the secretion of IL-5 and inducing eosinophilic airway inflammation through HLA-DQ (Papouchado et al., 2001). The epitope from Ppb of *P. bivia* was similar to the dust mite allergen dermatophagoides farinae 2 (Der f 2). As a common allergen in asthma, Der f 2 can be presented to T cells through HLA-DQ or HLA-DR, stimulating the production of IL-4 (Inoue et al., 1997; Lee et al., 2018). These findings suggested that the enrichment of these four microbial species in the children with asthma may activate the immune response through microbial mimicry peptides, thus involved in childhood asthma.

Through non-targeted metabolomics analysis of fecal samples, we found that lipid metabolism was significantly disturbed in the children with asthma. Lipid metabolism is a key driver of inflammation, and recent studies suggested that disruptions in lipid metabolism play an important role in the pathogenesis of asthma (Monga et al., 2020; Jiang et al., 2021). Through RDA analysis of the relationship between gut microbiota and differential metabolites in feces, it was found that bacteria such as *Prevotella* and *Bacteroides*, well known for their association with inflammation, may activate immunity by influencing lipid metabolites. *In vitro* experiments, it was demonstrated that *Prevotella* can induce dyslipidemia in HBE cells.

Currently, some mechanism behind the association between the disturbance of lipid metabolome and asthma has been proposed. One could be inflammatory responses induced by pro-inflammatory lipid metabolites such as CE, Cer and TG. It is well known that asthma is a chronic inflammatory disease. Dyslipidemia may induce a shift in the Th1/Th2 balance with a predominance of Th2 cells, and lower Th1/Th2 ratio in both mouse and human lymphocytes (Zhou et al., 1998; Matia-Garcia et al., 2021). There is a significant increase in CE, Cer and TG levels in in *P. bivia-treated* HBE cells. Hypercholesterolemia was found to be associated with high prevalence of asthma (Ko et al., 2018). Moreover, research found that elevated CE levels triggered the toll-like receptor signaling pathway in macrophages, thereby enhancing the immune response (Fessler and Parks, 2011). Cer plays a central role in sphingolipid metabolism, and their role in airway damage is controversial due to the complexity of their metabolic pathways. Lu et al. (2023) found that *Prevotella* can affect the prognosis of COVID-19 by modulating the sphingolipid metabolism pathway. Izawa et al. (2012) demonstrated that extracellular Cer interacted with CD300f (an immunoinhibitory receptor) limiting ovalbumin (OVA)-induced chronic airway inflammation. Sakae et al. (2023) indicated that Cer inhibit goblet cell hyperplasia in the airways induced by OVA. These findings suggested that Cer may act as anti-asthmatic lipids. However, James et al. (2021) found that pulmonary Cer trigger the recruitment of neutrophils, exacerbating allergic asthma and the reduction of Cer synthesis resulted in a decrease in Th2 responses and relieving OVA-induced asthma (Shin et al., 2021). This may be attributed to different acyl chain lengths causing variations in the functionality of Cer (Grösch et al., 2012). TG was the most abundant lipid in the human body, and an increase in their levels can adversely affect overall health. A cross-sectional study involving Korean adolescents indicated that elevated TG levels in the blood could lead to chronic inflammation in children, resulting in airflow obstruction and an increased prevalence of asthma (Chanachon et al., 2022) and a significant correlation was observed between TG levels and FeNO (fractional exhaled nitric oxide) in the patients with asthma (Jiang et al., 2021). *In vitro* experiments have shown that TG induced proliferation of CD4+ T cells and significantly increased the gene and protein levels of IL-17 and IFN-γ, thereby promoting inflammatory responses (Tan et al., 2022).

**Figure 7:**
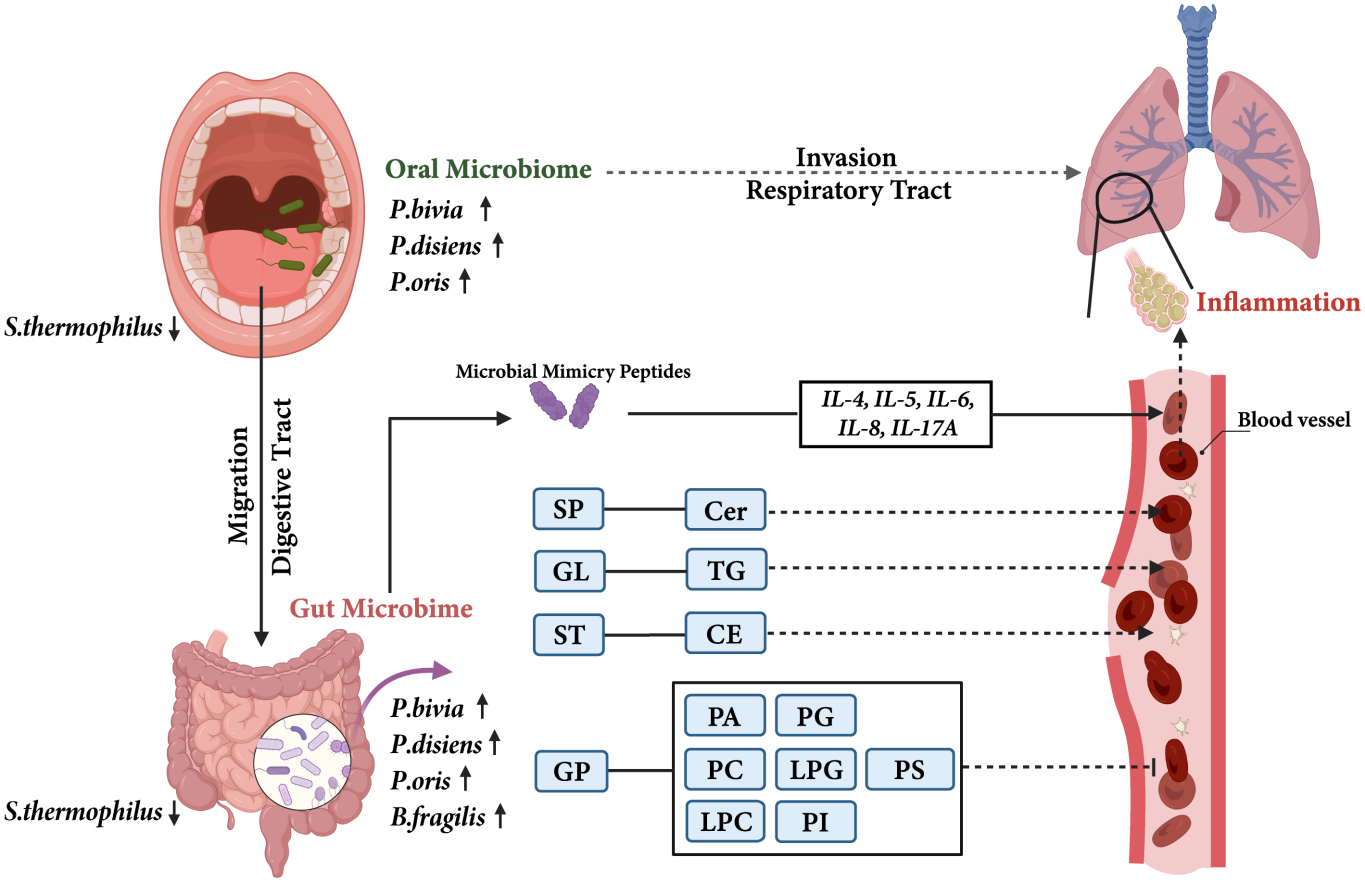
The potential mechanisms of oral-originated gut microbiota involvement in childhood asthma. The solid arrows indicated the results of this study, and the dashed arrows indicated previously reported results. SP, Sphingolipids; Cer, Ceramide; GL, Glycerolipids; TG, Triglyceride; ST, Sterol lipids; CE, Cholesterylesters; GP, Glycerophospholipids; PA, Phosphatidic acid; PC, Phosphatidylcholine; LPC, Lysophosphatidylcholine; PG, Phosphatidylglycerol; LPG, Lysophosphatidylglycerol; PI, Phosphatidylinositol; PS, Phosphatidylserine.

Another mechanism may be glycerophospholipids-involved in signaling and immune responses, which are major components of cell membranes and precursors of signaling molecules. They are secreted into the alveoli by airway epithelial cells to reduce alveolar surface tension and block pathogen invasion. Studies have shown that the decreasing of glycerophospholipids are involved in the pathogenesis of lung infections, asthma, and COPD (Mander et al., 2002; Gai et al., 2019). What’s more, we also observed a significant decrease in glycerophospholipid metabolites in the children with asthma. As a degradation product of PC, LPC can exert anti-inflammatory effects by reducing leukocyte extravasation, downregulating the formation of pro-inflammatory mediators IL-5 and IL-6, and upregulating the expression of anti-inflammatory mediators IL-4 and IL-10 (Hung et al., 2012; Monga et al., 2020). LPG is a precursor for *de novo* synthesis of PG which plays an important role, especially, in lung (Greenough, 1998). PG was a component of pulmonary surfactant that could reduce the surface tension at the alveolar air-liquid interface and exhibited immunomodulatory properties (Klein et al., 2020). PA can be degraded into LPA and pre-treatment with LPA has been shown to reduce the IL-13-induced STAT6 phosphorylation and cytokine release, accordingly exerting anti-inflammatory effects (Zhao and Natarajan, 2013). PI can increase airway hyperresponsiveness in asthma patients (Hallstrand et al., 2013). Stokes et al. (2016) found that liposomes containing PS could inhibit the inflammatory response induced by human rhinovirus, a major factor in acute exacerbations of chronic airway diseases. Therefore, based on these results, we postulated the potential mechanisms of oral-originated gut microbiota involved in childhood asthma (Figure 7).

This study has some limitations. First, the present study was a pilot study and had a small sample size. Second, due to the depth of metagenomic sequencing, homology analysis between oral microbiota and gut microbiota was only calculated by genetic distance and cannot fully represent the comprehensive relationship. Further research should be carried out to establish a direct causal relationship between oral-originated gut species and childhood asthma in multi-center samples by deep sequencing.

In conclusion, our study provided evidence that oral-originated microbiota in the gut, by secreting microbial mimicry peptides and altering in lipid metabolism, can influence childhood asthma. These findings highlighted the potential role of the oral-gut microbial axis in understanding the pathogenesis of asthma and metabolomic analysis of lipid profiles may give a new strategy for new biomarkers and new drug targets for asthma.

## Data availability statement

The datasets presented in this study can be found in online repositories. The names of the repository/repositories and accession number(s) can be found here: https://www.ncbi.nlm.nih.gov/, PRJNA1086671.

## Ethics statement

The studies involving humans were approved by Nanjing Medical University Clinical Research Ethics Committee under the reference number (2019)02080-1. The studies were conducted in accordance with the local legislation and institutional requirements. Written informed consent for participation in this study was provided by the participants’ legal guardians/next of kin.

## Author contributions

TY: Data curation, Formal analysis, Writing – original draft, Visualization, Validation, Methodology, Investigation. YB: Data curation, Formal analysis, Methodology, Writing – original draft. SC: Formal analysis, Writing – original draft. PJ: Formal analysis, Writing – original draft. ZZ: Writing – original draft, Formal analysis. LL: Resources, Writing – original draft. YK: Conceptualization, Funding acquisition, Project administration, Supervision, Writing – review & editing. QW: Conceptualization, Funding acquisition, Project administration, Resources, Supervision, Writing – original draft, Writing – review & editing.

## Footnotes

^1^ http://drive5.com/uparse/

^2^ http://rdp.cme.msu.edu/

^3^ www.i-sanger.com

^4^https://github.com/OpenGene/fastp, version 0.20.0.

^5^ cloud.majorbio.com

^6^https://github.com/voutcn/megahit, version 1.1.2.

^7^ http://metagene.cb.k.u-tokyo.ac.jp/

^8^http://www.diamondsearch.org/index.php, version 0.8.35.

^9^ http://www.iedb.org/

^10^ http://www.hmdb.ca/

^11^ http://www.metware.cn/

## Glossary

HMP: Human microbiome program
SCFAs: Short-chain fatty acids
BMI: Body mass index
PBMCs: Peripheral blood mononuclear cells
OTUs: Operational taxonomic units
VIP: Variable importance in the projection
RSD: Relative standard deviation
PCoA: Principal co-ordinates analysis
QC: Quality control
PCA: Principal component analysis
LDA: Linear discriminant analysis
LEfSe: LDA effect size
HBE: Human bronchial epithelial
RDA: Redundancy analysis
Der f 2: Dermatophagoides farinae 2
FeNO: Fractional exhaled nitric oxide
CE: Cholesterylesters
Cer: Ceramide
TG: Triglyceride
LPC: Lysophosphatidylcholine
LPG: Lysophosphatidylglycerol
PA: Phosphatidic acid
PC: Phosphatidylcholine
PG: Phosphatidylglycerol
PI: Phosphatidylinositol
PS: Phosphatidylserine
SDA: Stearidonic acid
EPA: Eicosapentaenoic acid
DG: Diglyceride
MG: Monoglyceride
TC: Total cholesterol
FA: Fatty Acyl
GL: Glycerolipids
GP: Glycerophospholipids
SP: Sphingolipids
