# Supplementary material for: The investigation of the role of oral-originated Prevotella-induced inflammation in childhood asthma
Source: Front Microbiol. 2024 May 28;15:1400079. doi: 10.3389/fmicb.2024.1400079 (PMC11165567; doi:10.3389/fmicb.2024.1400079)
Supplement: Supplementary file 1 [file Data_Sheet_1.docx]

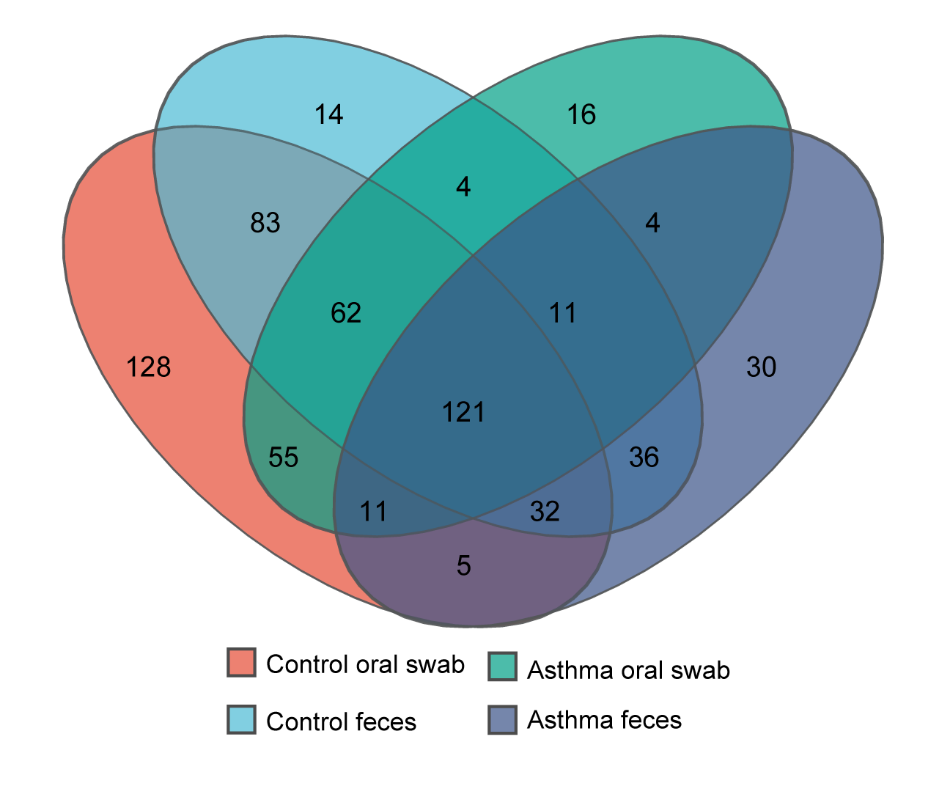


**Figure S1** **Venn diagrams showing the unique and shared OTUs among the different types of samples**


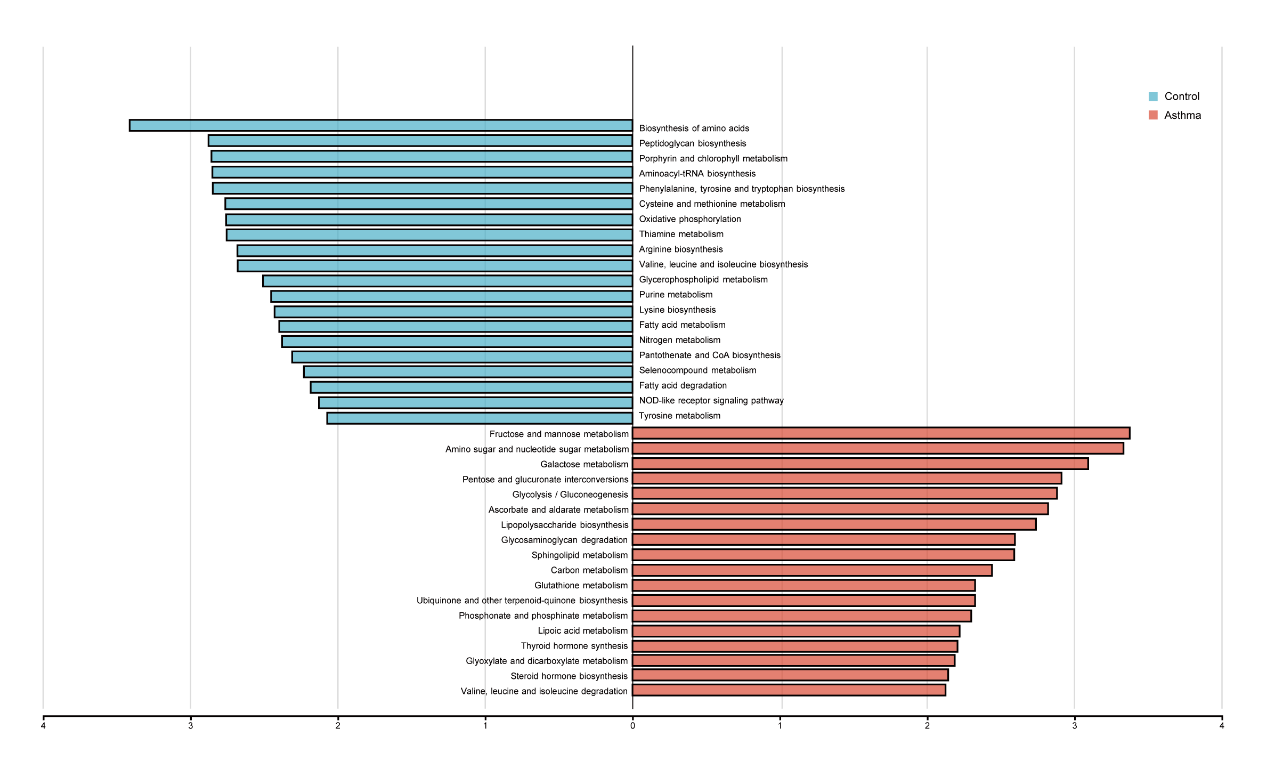


**Figure S2 Alterations of the gut microbiota function in children with asthma.**

Linear discriminant analysis (LDA) effect size (LEfSe) analysis determined distinct microbial functions associated with different groups based on KEGG pathways (LDA score > 2).

**
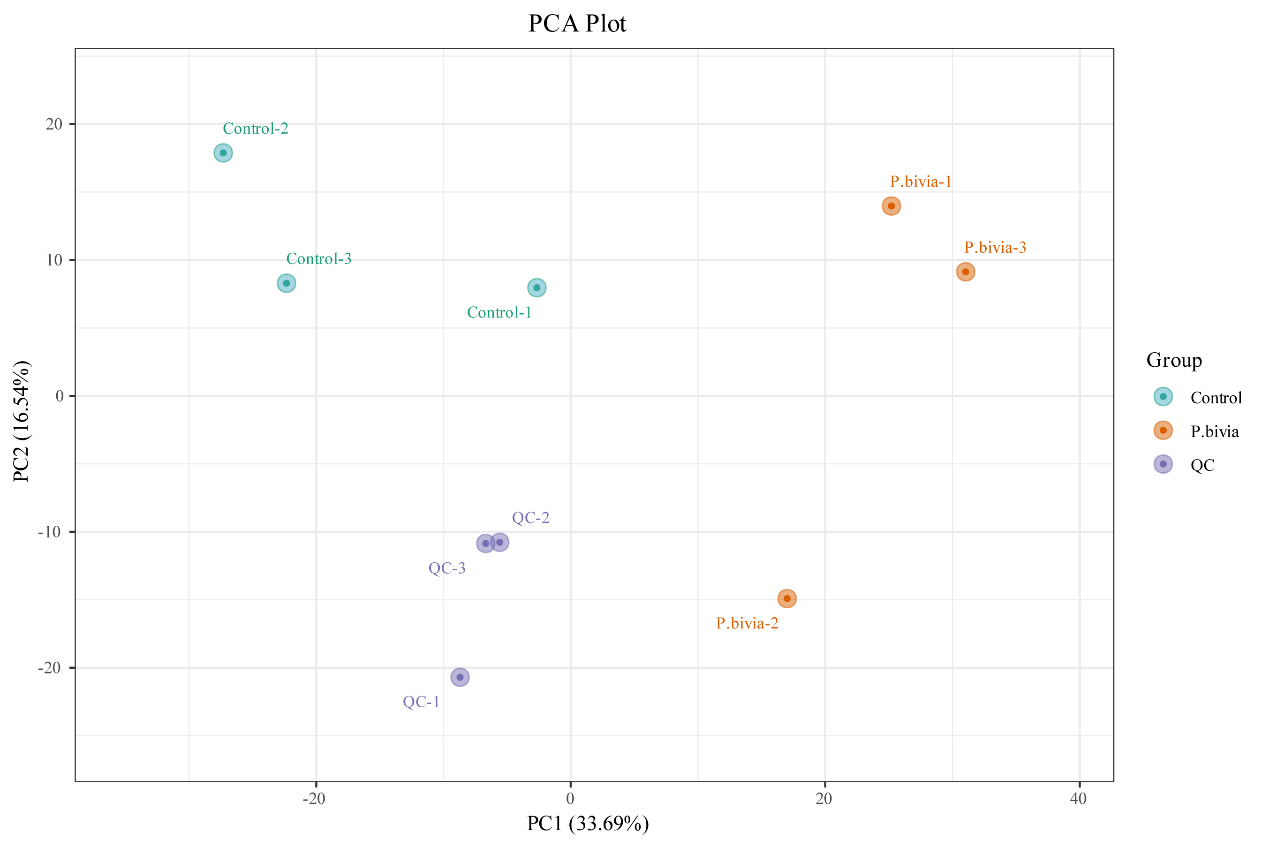
Figure S3 The PCA analysis of lipid metabolism in samples from each group**


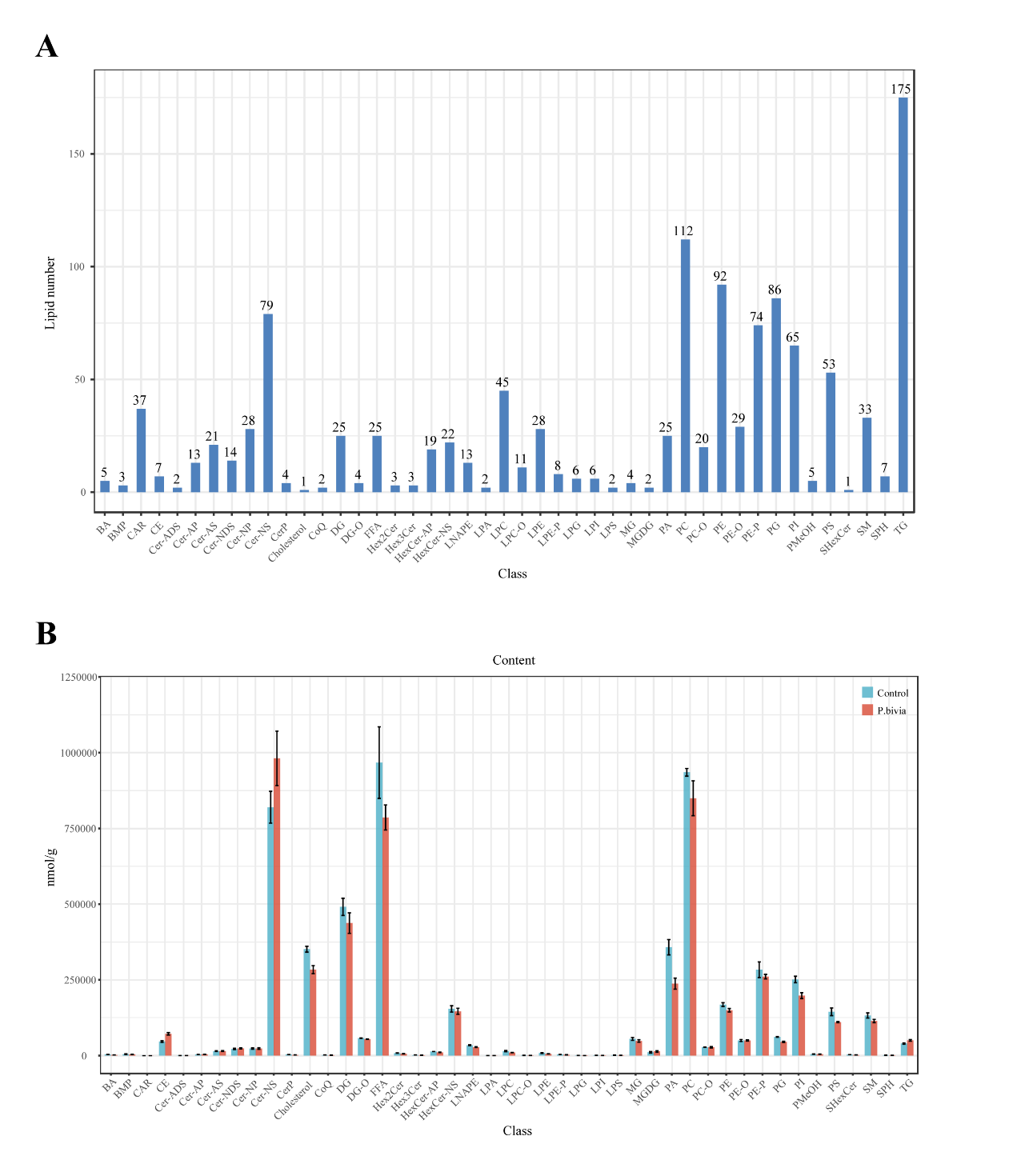
**Figure S4 The changes of lipid subclasses**

A: The distribution of lipid metabolites. B: The changes of the relative abundance of lipid subclasses.


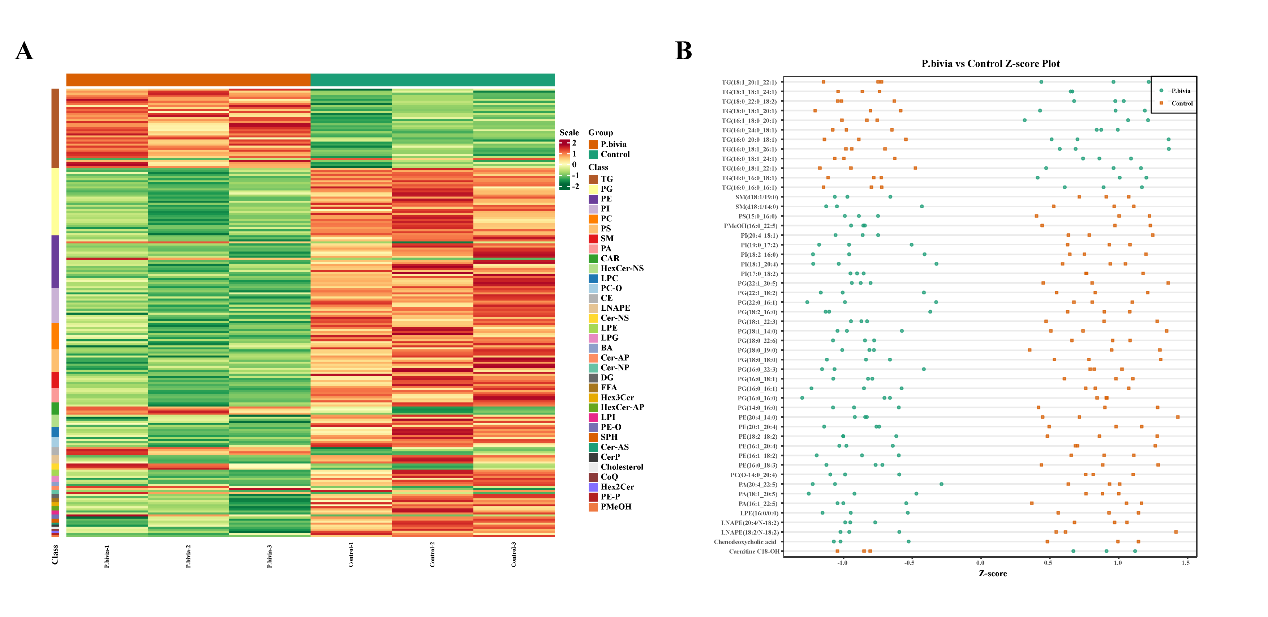


**Figure S5 The distribution of differential lipids among the control and the treated group**

A: The cluster heatmap of differential lipids. B: The Z-score plot of differential lipid metabolites.

**
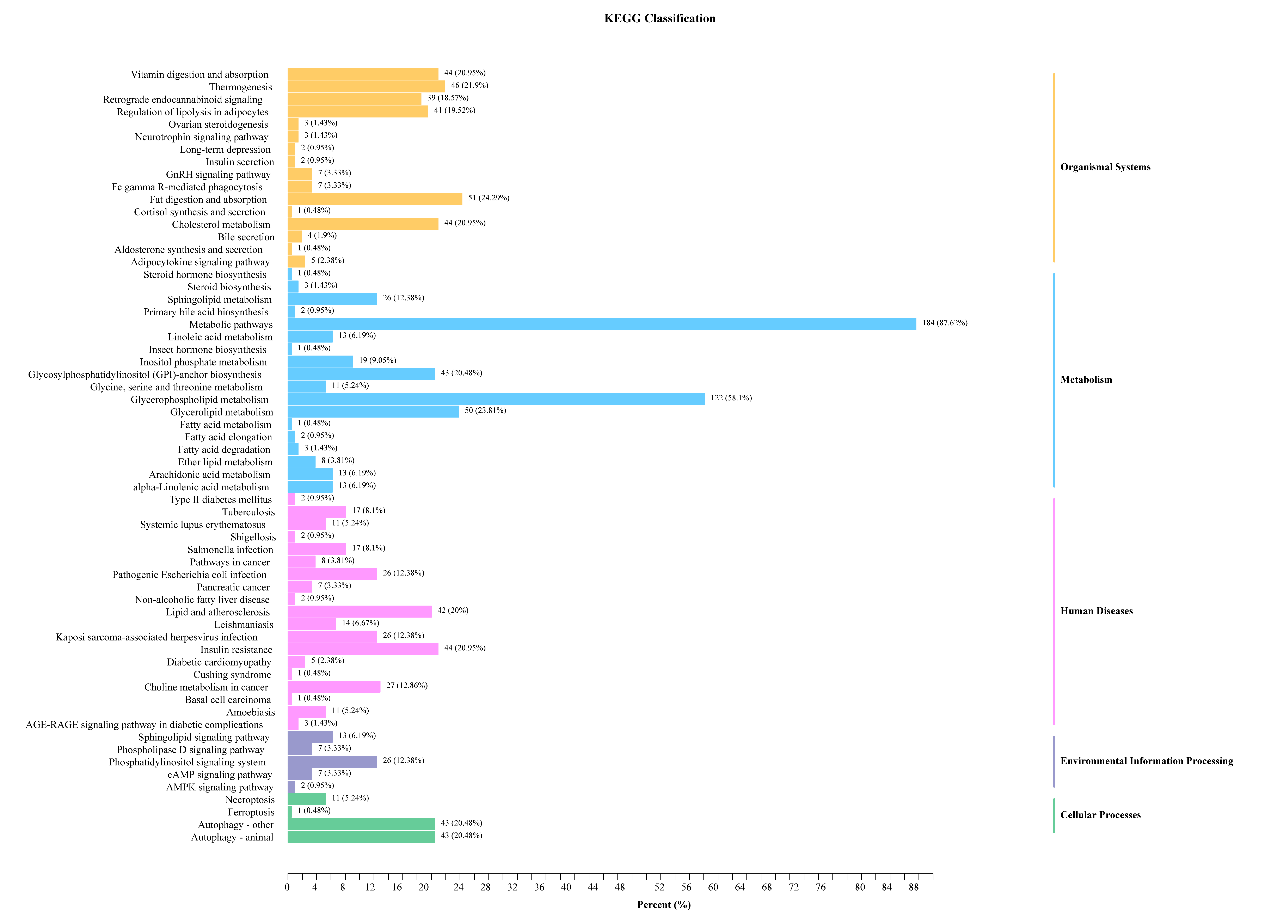
Figure S6 The KEGG classification plot of lipid metabolites**

**Table S1** **The demographic information of the children with asthma**

| **Number** | **Age (year)** | **Sex** | **Genetic Information** | **Allergen** |
| --- | --- | --- | --- | --- |
| Asthma 1 | 5.5 | Male | No family history | — |
| Asthma 2 | 7.5 | Male | No family history | molds |
| Asthma 3 | 5.1 | Female | No family history | dust mites |
| Asthma 4 | 10.1 | Male | Mother has asthma and hives | — |
| Asthma 5 | 5 | Female | No family history | — |
| Asthma 6 | 5.5 | Female | Mother and grandmother have a history of asthma | dog dander, egg white, milk |
| Asthma 7 | 7.4 | Female | Mother and elder brother have allergic rhinitis | dust mites, animal dander |
| Asthma 8 | 6.2 | Female | Father has allergic rhinitis | — |
| Asthma 9 | 7.8 | Male | No family history | — |
| Asthma 10 | 6.9 | Female | No family history | molds |
| Asthma 11 | 7.1 | Female | Mother has allergic rhinitis | — |
| Asthma 12 | 13.3 | Female | No family history | dust mites |
| Asthma 13 | 5.5 | Female | No family history | dust mites |
| Asthma 14 | 3 | Male | No family history | — |
| Asthma 15 | 9.3 | Female | Mother has a history of eczema | dust mites, molds |
| Asthma 16 | 1.4 | Male | No family history | — |
| Asthma 17 | 3.8 | Male | No family history | dust mites, molds, egg white, milk |
| Asthma 18 | 6 | Male | Mother is allergic to penicillin and cephalosporins | — |
| Asthma 19 | 7 | Male | Mother has a history of eczema | — |
| Asthma 20 | 8 | Male | No family history | dust mites |
| Asthma 21 | 3.8 | Female | No family history | dust mites |
| Asthma 22 | 2.3 | Male | No family history | dust mites, animal dander, molds, pollen |
| Asthma 23 | 7.1 | Female | No family history | — |
| Asthma 24 | 3.6 | Male | Mother has allergic rhinitis | — |
| Asthma 25 | 5.9 | Female | No family history | dust mites |
| Asthma 26 | 8.8 | Female | No family history | — |
| Asthma 27 | 6.8 | Male | No family history | seafood |
| Asthma 28 | 7 | Female | Mother has allergic rhinitis | — |
| Asthma 29 | 6.9 | Female | Mother has allergic rhinitis | mango |
| Asthma 30 | 12 | Male | No family history | — |

“—”: No testing.

**Table S2 Internal standard information in lipid metabolome**

| **Metabolites** | **CAS** | **Ion mode** | **m/z** | **RT (min)** |  |  |  |  |
| --- | --- | --- | --- | --- | --- | --- | --- | --- |
| PC (16:0/16:0)-d9 | 77165-56-1 | [M+COOH]- | 787.630236 | 6.52 |  |  |  |  |
| CAR (16:0)-d3 | 202480-73-7 | [M+H] + | 403.3 | 2.24 |  |  |  |  |
| CE (18:1)-d7 | 1416275-35-8 | [M+NH4] + | 675.67761 | 13.31 |  |  |  |  |
| Cer (d18:1/15:0)-d7 | — | [M+H] + | 531.559044 | 6.03 |  |  |  |  |
| Cer (d18:1-d7/16:0) | — | [M+H] + | 545.6 | 6.66 |  |  |  |  |
| Cer (d18:1-d7/18:0) | — | [M+H] + | 573.6 | 7.57 |  |  |  |  |
| Cer (d18:1-d7/24:0) | 1840942-15-5 | [M+H] + | 657.7 | 10.39 |  |  |  |  |
| Cer (d18:1-d7/24:1) | 54164-50-0 | [M+H] + | 655.7 | 10.19 |  |  |  |  |
| CoQ10-d9 | 303-98-0 | [M+H] + | 872.8 | 11.01 |  |  |  |  |
| GCDCA-d4 | 1201918-16-2 | [M-H] | 452.6 | 1 |  |  |  |  |
| LPA (17:0) | 799268-66-9 | [M-H] | 423.235515 | 2.62 |  |  |  |  |
| LPC (15:0)-d5 | 2342574-95-0 | [M+H] + | 487.4 | 1.9 |  |  |  |  |
| LPC (16:0)-d31 | 327178-91-6 | [M+H] + | 527.3 | 2.3 |  |  |  |  |
| LPC (17:0)-d5 | 2342575-12-4 | [M+H] + | 515.395091 | 3 |  |  |  |  |
| LPC (18:1-d7) | 2097561-13-0 | [M+H] + | 529.410741 | 2.57 |  |  |  |  |
| PA (17:0/17:0) | 154804-54-3 | [M-H] | 675.496482 | 7.35 |  |  |  |  |
| PC (15:0/18:1(d7)) | 2097561-16-3 | [M+COOH]- | 797.614586 | 6.22 |  |  |  |  |
| PC (16:0-d31/18:1) | 179093-76-6 | [M+H] + | 790.8 | 6.68 |  |  |  |  |
| PC (14:0/14:0)-d9 | 71479-88-4 | [M+COOH]- | 731.567636 | 5.44 |  |  |  |  |
| PE (15:0/18:1(d7)) | 2097561-15-2 | [M-H] | 709.562156 | 6.37 |  |  |  |  |
| PE (17:0-22:4)-d5 | 2342575-44-2 | [M-H] | 785.593456 | 6.98 |  |  |  |  |
| PG (15:0/18:1(d7)) | — | [M-H] | 740.556737 | 5.87 |  |  |  |  |
| PG (16:0/d31/18:1) | 327178-87-0 | [M-H] | 778.5 | 6.12 |  |  |  |  |
| PI (16:0-d31/18:1) | 799812-61-6 | [M-H] | 865.7 | 5.96 |  |  |  |  |
| PS (15:0/18:1(d7)) | — | [M-H] | 753.551986 | 5.82 |  |  |  |  |
| PS (16:0(d31)/18:1) | 327178-96-1 | [M-H] | 791.5 | 6.06 |  |  |  |  |
| PS (16:0/16:0)-d9 | 28152 | [M-H] | 743.567636 | 6.13 |  |  |  |  |
| TG (14:0/16:1/14:0)-d5 | 944709-23-3 | [M+NH4] + | 771.731589 | 10.87 |  |  |  |  |
| TG (16:0-15:1-16:0)-d5 | 2342574-85-8 | [M+NH4] + | 813.778539 | 11.43 |  |  |  |  |
| TG (17:0/17:1/17:0)-d5 | 958760-74-2 | [M+NH4] + | 869.8 | 12.38 |  |  |  |  |

m/z: mass-to-charge ratio; RT: retention time.

**Table S3 Information about microbial mimicry peptides**

| **Name** | **Autoantigen** | **Animo Acid Sequence** | **HLA restriction** | **References** | **Microbial antigen sequence** | **Microbiota** | **Consistency (%)** |
| --- | --- | --- | --- | --- | --- | --- | --- |
| Ppb | Der f 2 allergen（HDM) | PNKSKLGANAILGVS | HLA-DQ/HLA-DR | DOI: 10.1159/000237694 | PTKSKLGANAILGVS | *Prevotella bivia* | 93.33 |
| Rpd | Blattella germanica protein | RVALENAASVSGMLL | HLA-DQ | DOI: 10.4049/jimmunol.167.8.4627 | RVALENAASIAGMFL | *Prevotella disiens* | 80 |
| Lpo | Blattella germanica protein | LKVAAIKAPGFGDRR | HLA-DQ |  | LKICAVKAPGFGDRR | *Prevotella disiens/oris* | 80 |
| Fbf | Blattella germanica protein | WSKVVIAYEPVWAIG | HLA-DQ |  | FSKIVLAYEPVWAIG | *Bacteroides fragilis* | 85.71 |

**Table S4 Differential metabolites in lipid metabolism between the control and the group with asthma**

| Classify | Name | t_R_ | m/z | p-value | Fold change | VIP | polarity |
| --- | --- | --- | --- | --- | --- | --- | --- |
| Fatty Acyl (FA) | Malic acid | 1.069718 | 135.0282 | 0.003522 | 0.477886 | 1.46203 | positive |
|  | Caprylic acid | 6.384883 | 145.1227 | 0.000588 | 1.807751 | 1.55842 | positive |
|  | L-Carnitine | 0.803601 | 162.1130 | 0.022143 | 3.346817 | 1.0846 | positive |
|  | Dodecatrienoic acid | 7.10455 | 195.1385 | 0.001788 | 0.238557 | 1.41808 | positive |
|  | trideca-2,4,6-trienoic acid | 8.93715 | 209.1543 | 0.004815 | 0.457699 | 1.39115 | positive |
|  | tetradeca-2,4,6-trienoic acid | 7.86615 | 223.1700 | 0.004724 | 0.404338 | 1.30005 | positive |
|  | SDA | 6.270767 | 227.1281 | 0.010458 | 0.396825 | 1.378 | positive |
|  | 9,12-Octadecadiynoic acid | 9.096017 | 277.2172 | 0.0078 | 0.050435 | 1.16692 | positive |
|  | ALA | 8.98075 | 279.2328 | 0.003304 | 0.170376 | 1.26839 | positive |
|  | 9-HOTE | 8.312733 | 295.2280 | 0.026622 | 0.074129 | 1.01594 | positive |
|  | 12-Hydroxy-8,10-octadecadienoic acid | 9.400183 | 297.2433 | 0.032656 | 0.114637 | 1.01394 | positive |
|  | 13-HODE | 8.30715 | 297.2435 | 0.004461 | 0.269431 | 1.32398 | positive |
|  | EPA | 8.795933 | 303.2332 | 0.022559 | 0.137684 | 1.15079 | positive |
|  | Pimelylcarnitine | 7.239217 | 304.1756 | 0.039596 | 0.219447 | 1.08209 | positive |
|  | Icosa-5,8,11-trienoylcarnitine | 8.092392 | 391.2858 | 0.007606 | 0.053294 | 1.21538 | positive |
|  | 13-Docosenamide | 11.31963 | 338.3429 | 0.002236 | 1.724905 | 1.51687 | positive |
|  | Docosanamide | 11.92517 | 340.3583 | 0.000182 | 3.609403 | 1.69218 | positive |
| Glycerolipids (GL) | MG (0:0/PGD2/0:0) | 6.998383 | 427.2687 | 0.000771 | 0.277985 | 1.66839 | positive |
|  | DG (14:1(9Z)/22:6(4Z,7Z,10Z,13Z,16Z,19Z)/0:0) | 11.44875 | 611.4681 | 0.018074 | 0.346796 | 1.72545 | positive |
|  | DG (21:0/0:0/PGJ2) | 12.86837 | 717.5664 | 0.024334 | 0.273334 | 1.33371 | positive |
| Glycerophospholipids (GP) | LysoPC (14:1(9Z)/0:0) | 6.099967 | 466.2938 | 0.000588 | 0.353394 | 1.67948 | positive |
|  | LysoPC (14:0/0:0) | 6.213 | 468.3089 | 6.65E-05 | 0.260503 | 1.83236 | positive |
|  | PC (18:1(12Z)-2OH (9,10)/2:0) | 6.347167 | 596.3538 | 0.027686 | 0.044356 | 1.10935 | positive |
|  | PC (2:0/5-iso PGF2VI) | 7.078283 | 608.3188 | 0.028077 | 0.298902 | 1.26577 | positive |
|  | PA (15:0/18:0) | 12.48947 | 663.4944 | 0.018728 | 0.447321 | 1.13982 | positive |
| Sphingolipids (SP) | Cer(d18:0/20:3(6,8,11)-OH (5)) | 13.49633 | 606.5438 | 0.04121 | 2.245257 | 1.52862 | positive |

m/z, mass to change ratio; tR, retention time; VIP, The variable importance in the projection; Fold Change: the asthmatic group to the control group.
